# The Role of Surgical Lung Biopsy in Diagnosis and Treatment Guidance for Interstitial Lung Diseases: A Single-Center Retrospective Study

**DOI:** 10.3390/jcm15103956

**Published:** 2026-05-20

**Authors:** Melike Ülker, Barış Demirkol, Ramazan Eren, Dilekhan Kizir, Celal Buğra Sezen, Volkan Erdoğu, Muzaffer Metin, Erdoğan Çetinkaya

**Affiliations:** 1 Department of Thoracic Surgery, Yedikule Chest Diseases and Thoracic Surgery Training and Research Hospital, Istanbul 34020, Türkiye; 2 Department of Chest Diseases, Yedikule Chest Diseases and Thoracic Surgery Training and Research Hospital, Istanbul 34020, Türkiye

**Keywords:** interstitial lung disease, surgical biopsy, VATS

## Abstract

**Purpose:** Interstitial lung disease encompasses a heterogeneous group of disorders requiring subgroup-specific treatment strategies. Surgical lung biopsy is recommended in patients who remain diagnostically unclassified despite clinical and radiological evaluation. This study aimed to evaluate the diagnostic yield, postoperative outcomes, and therapeutic impact of surgical lung biopsy in patients with interstitial lung disease. **Methods:** Cases of surgical biopsy performed for interstitial lung disease between 2018 and 2023 were retrospectively analyzed. All patients underwent a comprehensive preoperative evaluation, including HRCT, pulmonary function testing with DLCO analysis, bronchoscopy, and multidisciplinary assessment, before surgical biopsy was considered for diagnostically unclassified cases. Postoperative complications, mortality rates, definitive diagnoses, and treatments were evaluated. **Results:** A total of 253 patients were included in the study, comprising 150 males (59.3%) and 103 females (40.7%). The mean age was 58.4 ± 12.5 years. Minor complications were observed in 14 cases (5.5%), most commonly prolonged air leakage, while major complications occurred in 7 cases (2.8%), including hemorrhage requiring revision surgery and postoperative respiratory failure. Mortality was reported in two cases (0.8%). Comparison between surgical approaches demonstrated statistically significant differences regarding postoperative complication rates and drainage duration (*p* = 0.008, *p* < 0.001). All patients who received a definitive diagnosis were initiated on disease-specific treatments. Medical treatment was initiated for 197 cases (77.8%). Specific treatment was started for 174 cases (68.8%) following the determination of an interstitial subgroup diagnosis. **Conclusions:** Surgical lung biopsy performed via VATS is a safe procedure that provides significant diagnostic and therapeutic benefit in patients with interstitial lung disease, particularly in diagnostically unclassified cases despite multidisciplinary evaluation.

## 1. Introduction

Surgical lung biopsy remains an important diagnostic tool in selected patients with interstitial lung disease who remain diagnostically unclassified despite comprehensive clinical, radiological, and bronchoscopic evaluation. Recent studies and international guidelines continue to support the role of surgical lung biopsy in multidisciplinary diagnostic assessment, particularly in cases with uncertain clinical and radiological findings [1].

Most previous studies showed that either an open lung biopsy with thoracotomy or video-assisted thoracoscopic surgery (VATS) approaches are appropriate for these patients for a definitive diagnosis. The outcomes of postoperative complications following surgical lung biopsy appear unclear. Although both procedures are often performed under general anesthesia, VATS lung biopsy has potential advantages compared to thoracotomy such as less postoperative pain and hospital stay, better cosmetic outcome, immune system and quality of life [2,3]. In addition, morbidity and mortality rates are favorable in patients undergoing VAT procedures. The morbidity rate ranges between 5.8% and 14.7%, and the mortality rate varies between 1.4% and 4% [4,5].

At the same time, accurate pathological subgroup classification through surgical lung biopsy plays an important role in determining disease-specific treatment strategies in interstitial lung disease. In the study conducted by Richeldi et al., the use of antifibrotic agents such as nintedanib and pirfenidone was shown to reduce the decline in FVC by nearly half compared with the placebo group. Therefore, obtaining a definitive diagnosis with VATS surgical lung biopsy may directly contribute to appropriate treatment selection and individualized patient management [6].

We conducted a retrospective study to reveal the clinicopathological features of patients who underwent surgical lung biopsy and analyzed the postoperative complications, morbidity and mortality rates for various diagnostic procedures.

## 2. Methods

A total of 253 patients underwent either mini-thoracotomy or video-thoracoscopy for definitive diagnosis of interstitial lung disease between 2018 and 2023 in our department. All patients underwent investigations for interstitial lung disease before the operation, including radiological imaging, bronchoscopy, and bronchoalveolar lavage, without any definitive diagnosis.

We analyzed the clinicopathological features of the patients, including age, gender, smoking history, comorbidities, surgical procedures, histological examination, drainage time, postoperative complications, morbidity, mortality rates, and postoperative treatment.

All patients presenting with various symptoms were requested to undergo high-resolution computed tomography (HRCT), spirometry (SFT), diffusing capacity for carbon monoxide (DLCO) testing, and connective tissue marker analysis. They were evaluated in an interstitial council with the participation of pulmonology, occupational diseases, and rheumatology specialists. Cases that could not be diagnosed following the council evaluation were further assessed by the pulmonology clinic using bronchoscopy, bronchoalveolar lavage, and cell counting analysis. Cases that remained undiagnosed after these interventional procedures were evaluated in a multidisciplinary council involving pulmonology, thoracic surgery, radiology, and pathology specialists. The side of the operation and the anatomical region for wedge resection were determined. In cases with widespread involvement, obtaining one sample each from the upper and lower lobes is our routine procedure. Cases with a thoracic tube in place for more than five days were classified as having prolonged air leakage.

The surgical procedure was performed using uniportal/biportal VATS in 208 cases (82.2%), while a subxiphoid approach was applied in 37 cases (14.6%). Mini-thoracotomy was conducted in 8 cases (3.2%) due to extensive pleural adhesions.

### 2.1. Surgical Approach

**Figure 1:**
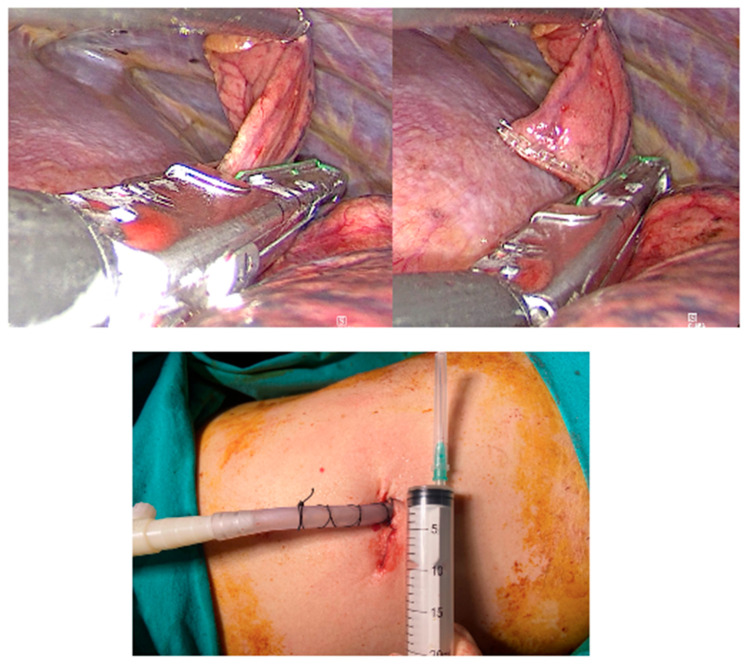
Perioperative and postoperative images.

The patients were positioned in a lateral decubitus position, and soft silicone pillows were used to ensure the patients were in a comfortable position. First trocar access was generally placed at the eighth intercostal space along the midaxillary line for a 30-degree camera. The second trocar access, which was a 1 cm skin incision, was performed along the anterior and superior area from the camera port. The entry sites used for the uniportal or biportal technique were the same, while for the subxiphoid technique, an incision was made in the subxiphoid region. Radiologically and much more macroscopically, lung biopsies were performed as two wedge resections from the upper and lower lobes (Figure 1). Resected specimens were taken out of the anterior port. A 24 or 28-ch chest tube was inserted from the lowest incision after hemostasis and aerostasis were carefully checked. At the end of the procedure, lung re-expansion was attained with the checking of thoracoscopic vision.

Mini-thoracotomy was defined as a limited enlargement of the utility incision during minimally invasive surgery due to dense pleural adhesions or technical intraoperative difficulties. In patients undergoing mini-thoracotomy, intercostal closure was routinely performed using a pericostal suture technique to optimize chest wall restoration and minimize postoperative pain.

### 2.2. Postoperative Process

In our clinical practice, disease exacerbation in interstitial lung disease was defined as worsening dyspnea, newly developed or increased oxygen requirement, acute deterioration in respiratory symptoms, and newly developed radiological infiltrates not explained by cardiac failure, infection, or fluid overload. Surgical lung biopsy is deferred in patients who are experiencing an active exacerbation of their interstitial lung disease. Surgical intervention is planned in close collaboration with the pulmonology department and is scheduled only when deemed appropriate by the attending pulmonologists. In the postoperative period, if a patient experiences disease exacerbation, treatment decisions are made in coordination with pulmonologists to ensure individualized and optimal management.

Following discharge, the first postoperative surgical follow-up was routinely performed one week after surgery and included clinical assessment and chest radiography. A second outpatient follow-up was generally scheduled within 2–3 weeks after surgery, following the availability of the final pathological diagnosis. Patients with stable clinical and radiological findings were subsequently referred back to the pulmonology department for disease-specific treatment planning and follow-up.

Among patients who initiate specific therapy based on their pathological diagnosis, follow-up is conducted by pulmonology specialists, focusing on qualitative clinical parameters such as reduction in the number of exacerbations, improved six-minute walk distance, decreased oxygen requirement, and enhanced exercise tolerance. These patient-centered outcomes guide therapeutic adjustments more effectively than numeric pulmonary function parameters in routine practice.

### 2.3. Medical Treatment

In this section, we tried to summarize our clinical treatment. The first and most important step in the treatment of hypersensitivity pneumonitis is removing the patient from exposure to the causative factor. For patients whose symptoms persist despite the removal of the causative factor or who exhibit severe symptoms, immunosuppressive therapies, primarily corticosteroids, are administered as the first line of treatment. For patients exhibiting a progressive fibrotic course despite immunosuppressive therapy, non-fibrotic treatments are preferred.

In the treatment of Idiopathic Pulmonary Fibrosis (IPF), antifibrotic agents such as pirfenidone and nintedanib are used. Patients in advanced stages are referred for evaluation for lung transplantation. In patients diagnosed with Nonspecific Interstitial Pneumonia (NSIP), corticosteroids are initiated in cases where the inflammatory component is predominant, with additional immunosuppressive therapies administered if deemed necessary. In fibrotic NSIP cases, antifibrotic drugs are preferred for patients with a progressive fibrotic course, in addition to immunosuppressive therapy.

In patients diagnosed with organizing pneumonia, etiological evaluation is conducted first, followed by the administration of corticosteroid therapy in most cases. Post-treatment, patients are monitored for the risk of recurrence. In the treatment of sarcoidosis, the therapeutic plan is tailored based on the stage and symptoms of the disease. In addition to inhaled steroid therapy, oral corticosteroids are initiated in patients with appropriate indications. For patients requiring long-term immunosuppressive therapy, alternative treatment options such as methotrexate and azathioprine are employed.

In the treatment of cystic lung diseases, disease-specific therapeutic approaches are adopted. In the treatment of Pulmonary Langerhans Cell Histiocytosis (PLCH), smoking cessation is prioritized, and immunosuppressive agents are used in patients whose symptoms persist. For patients with Interstitial Pneumonia with Autoimmune Features (IPAF), immunosuppressive drugs are employed to suppress the immune response, with treatment plans individualized in collaboration with rheumatology support.

The therapeutic approach for unclassifiable interstitial lung diseases requires a multidisciplinary evaluation. In managing these diseases, personalized treatment plans are developed by considering the severity of the patient’s clinical symptoms, lung function test results, and disease progression. In treatment selection, immunosuppressive therapy is preferred for cases suspected to be immune-mediated based on multidisciplinary evaluation, while antifibrotic therapies are employed in cases where fibrotic findings are predominant.

Corticosteroid therapy was generally initiated with prednisone at doses ranging between 0.5 and 1 mg/kg/day according to disease severity and radiological progression. Treatment duration and tapering schedules were individualized based on clinical response, pulmonary function, and multidisciplinary evaluation.

For pulmonary malignancy cases, treatment options such as surgery, chemotherapy, or radiotherapy are determined based on the tumor type and stage following multidisciplinary evaluation in an oncology council. In patients with lung involvement due to rheumatological diseases, treatment is conducted under the supervision of the rheumatology department.

### 2.4. Statistical Analysis

While the data were analyzed retrospectively through patient files, there was no missing data from the patients in the study. Microsoft Office Excel 2020 and Word 2019 versions were used to create the database. The IBM SPSS Statistics Version 26 program was used for statistical calculations. The descriptive results of the study are presented together with the corresponding percentages in the case of nominal or ordinal variables. Continuous variables are presented with mean and standard deviation values. “*p*” value below 0.05 was considered significant.

The study was approved by the ethics/scientific committee of Tekirdag Dr. Ismail Fehmi Cumalıoglu City Hospital and was conducted in accordance with the principles of the Declaration of Helsinki, under the number 91/2024. Informed consent for participation was obtained from all subjects involved in the study.

## 3. Results

A total of 253 patients were included in the study, comprising 150 males (59.3%) and 103 females (40.7%). The mean age was 58.4 ± 12.5 years. Symptoms observed included shortness of breath in 225 cases (88.9%), cough in 128 cases (50.6%), chest discomfort in 13 cases (5.1%), sputum in 13 cases (5.1%), and hemoptysis in 1 case (0.4%), while 9 cases (3.6%) were asymptomatic. Of the cases, 175 (69.2%) were non-smokers. Among smokers, cigarette consumption averaged 28.7 ± 17.9 pack-years (range 5–100).

Comorbidities included diabetes mellitus in 39 cases (15.4%), hypertension in 26 cases (10.3%), and coronary artery disease in 17 cases (6.7%). Preoperative FEV1 was 2.03 ± 0.76 L (Range: 0.62–4.74), while preoperative FVC was 2.41 ± 0.88 L (Range: 0.68–4.98). The demographic characteristics of the cases are presented in Table 1.

The side of the operation was predominantly the right side in 203 cases (80.2%) and the left side in 50 cases (19.8%). The most common procedure performed was wedge resection from both the upper and lower lobes, applied in 204 cases (80.6%). In the preoperative evaluation using computed tomography, lymph node dissection was performed in 3 cases (1.2%) due to detected lymphadenopathy, and pleural biopsy was added in 2 cases (0.8%) due to pleural thickening or pleural irregularities. Other resection locations, as well as perioperative and postoperative variables, are shown in Table 2.

The mean drainage duration was 2.9 ± 1.5 days (Range: 1–15). Complications were observed in 22 cases (8.7%). Prolonged air leak was detected in 19 cases (7.5%), with five of these cases requiring additional drainage, while the remaining group was managed conservatively. One case underwent revision surgery due to hemorrhage, and ductus embolization was performed in one case due to chylothorax. Minor complications were observed in 14 cases (5.5%), while major complications occurred in 7 (2.8%). Postoperative complications were observed in 18.9% of patients in the VATS group, 18.9% in the subxiphoid group, and 25% in the mini-thoracotomy group. Comparison between surgical approaches demonstrated a statistically significant difference regarding postoperative complication rates (*p* = 0.008). Mean drainage duration was 2.8 ± 1.5 (R:1–15) days in the VATS group, 3.3 ± 1 (R:1–6) days in the subxiphoid group, and 3.5 ± 2.1 (R:2–8) days in the mini-thoracotomy group, which was also statistically significant (*p* < 0.001).

Mortality was reported in two cases (0.8%) during the study period. One patient was re-intubated due to hypoxia and respiratory failure and died of multiorgan failure. Another case died in the intensive care unit within the first 24 h postoperatively due to myocardial infarction. No mortality was observed in patients who underwent the mini-thoracotomy procedure.

**Figure 2:**
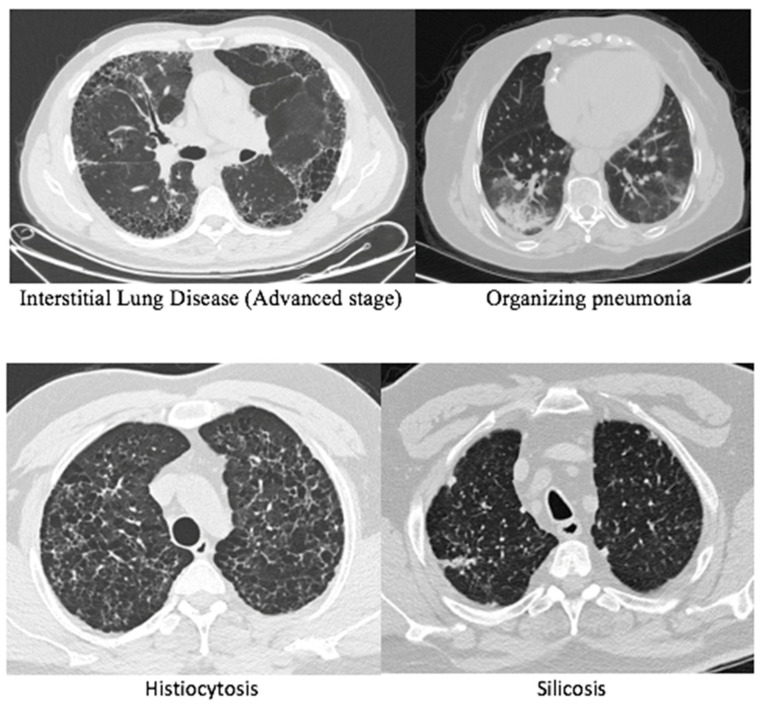
Images according to pathology diagnoses 1.

**Figure 3:**
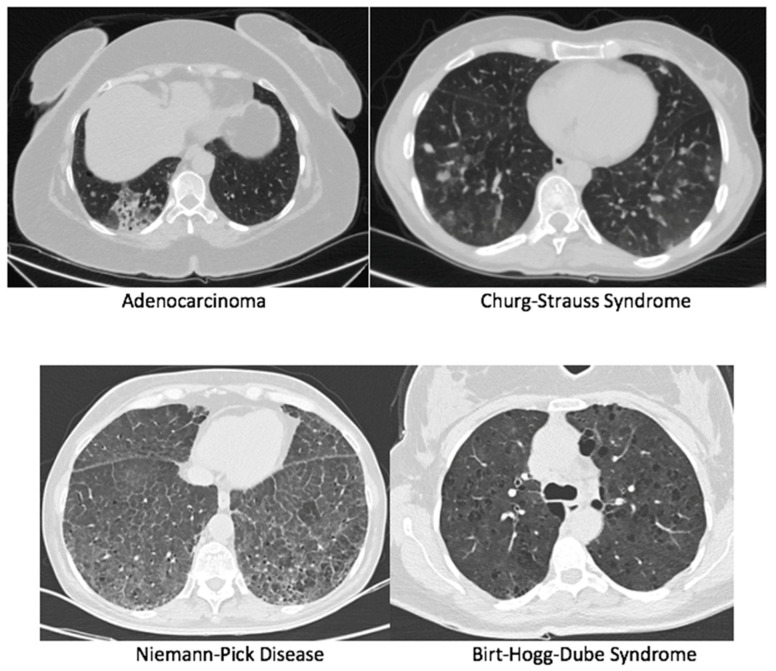
Images according to pathology diagnoses 2.

Pathological examination of specimens revealed hypersensitivity pneumonitis as the most common diagnosis in 111 cases (43.9%), followed by idiopathic pulmonary fibrosis in 45 cases (17.8%). There was one case each of Churg-Strauss syndrome, Niemann-Pick disease, and Brit–Hoge–Dube syndrome. Images of the cases according to pathology diagnoses are shown in Figure 2 and Figure 3. A definitive diagnosis could not be established in only 20 cases (7.9%). The definitive diagnosis rate was 92.1%. Other pathological classifications are detailed in Table 3. All patients who received a definitive diagnosis were initiated on disease-specific treatments. Medical treatment aimed at diagnosis was initiated for 197 cases (77.8%), while the remaining group was placed under follow-up. Specific treatments were started for 174 cases (68.8%) after the determination of an interstitial subgroup diagnosis. The treatment options included prednisone in 118 cases (46.6%), pirfenidone in 35 cases (13.8%), nintedanib in 23 cases (9.1%), and cellcept in 9 cases (3.6%).

## 4. Discussion

Interstitial lung disease is a broad-spectrum condition for which different treatments are applied based on subgroup diagnoses. Surgical biopsy plays a critical role in cases where clinical and radiological evaluations fail to establish a diagnosis or when the clinical course raises suspicion. While cryobiopsy has a high diagnostic yield, it has been available and actively used in our clinic since 2020. In a study conducted by Turan and colleagues in the pulmonary diseases department of our hospital, the diagnostic accuracy of transbronchial cryobiopsy was 66.6% based on pathology and 74.1% following multidisciplinary council evaluation [7].

In the study conducted by Lieberman et al. in 2017, 47 cases were evaluated, reporting a minor complication rate of 21.3%, a major complication rate of 6.4%, and a mortality rate of 8.5% [8]. In our study, the minor complication rate was 5.5%, the major complication rate was 2.8%, and the mortality rate was 0.8%. In our study, the minor complication rate was 5.5%, the major complication rate was 2.8%, and the mortality rate was 0.8%. Although our complication and mortality rates were numerically lower than those reported by Lieberman et al., direct superiority cannot be concluded because both studies included relatively limited patient populations. This study recommends performing a surgical biopsy in the early stages of the disease, typically when there is no widespread lung involvement, and symptoms are mild to moderate. In our clinic, cases in the exacerbation phase are not subjected to surgical biopsy. In the study by Lieberman et al., the evaluation of computed tomography scans by a thoracic radiologist showed only 60.5% concordance with the final pathological diagnosis. Among cases evaluated in a multidisciplinary council based on biopsy results, a definitive diagnosis could be established, and treatment changes were made in 51.1% of cases. One of the diagnostic groups of particular importance includes malignancy cases. In the study by Lieberman et al., 4.3% of cases were diagnosed with malignancy, whereas in our study, the malignancy diagnosis rate was 2.4%. The accurate diagnosis and treatment guidance of previously undiagnosed malignancy cases remain a critical aspect of care.

In the study conducted by Durheim et al. in 2017 [9], data from The Society of Thoracic Surgeons database were utilized, and 3085 cases were analyzed. Postoperative respiratory distress was observed in 2.9% of cases in the study, with a mortality rate of 1.5% [9]. In our study, respiratory failure was observed in one case (0.4%), which resulted in mortality. The study highlighted that preoperative corticosteroid use was identified as a risk factor for mortality. In our study, preoperative corticosteroid use was minimal, as most cases lacked a definitive diagnosis before surgery.

In the study conducted by Nguyen and Meyer, a literature review revealed that the minor complication rate was 18.2% for open biopsy and 9.6% for VATS biopsy, while mortality rates were 4.3% for open biopsy and 2.1% for VATS biopsy [10]. In our study, both complication and mortality rates were lower than these figures, with the VATS technique being utilized in our clinic. Due to severe pleural adhesions, mini-thoracotomy was performed in 8 cases (3.2%), with partial pneumolysis carried out to create a resectable area. No mortality was observed in the mini-thoracotomy group.

Previous studies have reported variable mortality rates following surgical lung biopsy in patients with interstitial lung disease, particularly in patients with advanced disease, impaired pulmonary reserve, or acute exacerbation. Park et al. identified advanced age, reduced pulmonary function, and severe postoperative respiratory complications as major risk factors associated with mortality following surgical lung biopsy [5]. Similarly, Durheim et al. reported that respiratory failure remains one of the most important causes of postoperative mortality after thoracoscopic lung biopsy in ILD patients [9]. Hutchinson et al. additionally emphasized that surgical lung biopsy should preferably be performed in experienced high-volume centers due to the potential perioperative risks associated with these complex patients [1]. In our study, the observed mortality rate was acceptable and comparable with the current literature, likely reflecting careful patient selection and multidisciplinary perioperative management.

In the study conducted by Otsuka et al. in 2022, 129 cases were analyzed, and 26.4% of the cases could not be classified based on their pathological diagnoses and were categorized as unclassifiable idiopathic interstitial pneumonia. As the number of biopsies performed on the same case increases, the rate of achieving a surgical diagnosis also rises; however, this also creates a predisposition for complications. While this study reported a rate of 9% for biopsies performed from three different lobes, in our study, this was performed in 3% of cases. In the study by Otsuka et al., the complication rate was found to be 10.1%, while no mortality was observed [11].

In a meta-analysis conducted by Han et al., 2148 cases from 23 studies were evaluated, and the median rate of diagnosis through surgical biopsy was reported as 95%. The diagnostic rate ranged from 42% to 100%, with only one study reporting a rate below 70%. When comparing six studies that applied both VATS and open surgery, no significant difference was observed in the determination of the pathological diagnosis. In the pathological specimen evaluation, idiopathic pulmonary fibrosis was the most common diagnosis at 33.5%, followed by NSIP at 11.9% and HP at 9.6% [12]. In our study, the rate of definitive diagnosis was 92.1%, and the most common diagnosis in the pathological specimen evaluation was hypersensitivity pneumonitis at 43.9%, followed by idiopathic pulmonary fibrosis at 17.8%.

In the study conducted by Fibla et al., 311 cases underwent surgical biopsy, and 74.6% achieved a specific, definitive diagnosis. The most common diagnoses were idiopathic pulmonary fibrosis at 39% and cryptogenic organizing pneumonia at 10%. In these cases, 77% started a new treatment, 40.7% had a change in the treatment strategy, and 6.8% discontinued the previous treatment. In our study, 77.8% of cases started medical treatment, while 68.8% of cases in the IAH subgroups began specific treatment [13]. It is evident that these studies provided therapeutic benefits for the disease through definitive diagnosis. The literature review and comparative analysis summarized in Table 4.

After clinical and radiological evaluation, the diagnostic rates through bronchoscopy and transbronchial cryobiopsy are high, and typical UIP cases in smokers can receive a diagnosis. In the pathology review of our study, hypersensitivity pneumonia was the most commonly observed condition, along with specific and rare subdiagnoses, indicating that the cases referred for surgery were optimally selected. Being a specialized center in pulmonology and thoracic surgery, and a tertiary hospital, with extended procedures being performed and a high annual patient intervention rate, this is an experienced institution. It is crucial to plan the diagnosis and treatment of specialized groups, such as those with interstitial lung diseases, through a multidisciplinary approach.

Recent advances in artificial intelligence and radiological pattern recognition may contribute to improved diagnostic accuracy and patient selection in interstitial lung disease. In the future, integration of artificial intelligence-based imaging analysis with multidisciplinary evaluation may further optimize the role of surgical lung biopsy in thoracic surgery practice.

## 5. Limitation

Transbronchial cryobiopsy has been actively used in our clinic in recent years. However, during the course of the study, it was not used equally throughout the periods due to technical issues. Although all patients who underwent surgical lung biopsy were included in the study, the total number of patients evaluated for interstitial lung disease during this process is unknown.

This study included a heterogeneous group of interstitial lung disease subtypes diagnosed through surgical biopsy. As a tertiary referral center specializing in thoracic surgery and pulmonology, our institution frequently receives diagnostically complex interstitial lung disease cases requiring multidisciplinary evaluation and surgical consultation. Therefore, referral-related selection bias may have influenced the relatively high diagnostic yield observed in this study. Although our center is a national reference unit with high procedural volume, the diversity of diagnoses limited standardized follow-up of pulmonary function across subgroups. Additionally, a prospective protocol for systematic pre- and post-biopsy pulmonary function testing was not implemented during the study period. Future studies focusing on specific diagnostic subgroups with structured functional follow-up are needed to better understand the impact of surgical lung biopsy on respiratory function.

## 6. Conclusions

Surgical lung biopsy remains an important diagnostic tool in selected patients with interstitial lung disease, particularly in cases that remain unclassified despite comprehensive multidisciplinary evaluation. In our study, VATS-guided surgical biopsy demonstrated acceptable morbidity and mortality rates while providing a high diagnostic yield and significant contribution to disease-specific treatment planning. Accurate pathological subgroup classification may directly influence individualized therapeutic strategies, including antifibrotic and immunosuppressive treatments. Careful patient selection and multidisciplinary decision-making remain essential for optimizing clinical outcomes in this complex patient population.

## Figures and Tables

**Table 1:** Clinical and demographic characteristics of the patients.

Variables	All Patients 253 (%)
Age ± SD (mean years)	58.4 ± 12.5
Male	150 (59.3)
Female	103 (40.7)
Non-smoker	175 (69.2)
Ex smoker	78 (30.8)
Smoking (pack/year) ± SD	28.7 ± 17.9 (R: 5–100)
FVC (L)	2.41 ± 0.88 (R: 0.68–4.98)
FEV1 (L)	2.03 ± 0.76 (R: 0.62–4.74)
FEV1/FVC (%)	82.15 ± 15.22 (R: 36–118)
Symptom	
None	9 (3.6)
Hemoptysis	1 (0.4)
Mucus	13 (5.1)
Chest discomfort	13 (5.1)
Cough	128 (50.6)
Shortness of breath	225 (88.9)
Comorbidity	
Diabetes Mellitus	39 (15.4)
Hypertension	26 (10.3)
Coronary Artery Disease	17 (6.7)
Chronic Obstructive Pulmonary Disease	7 (2.8)
History of Malignancy	5 (2)
Rheumatological Disease	3 (1.2)
Chronic Renal Failure	1 (0.4)

SD: Standard deviation, R: Range, FVC: Forced vital capacity, FEV1: Forced expiratory volume in the first second.

**Table 2:** Perioperative and postoperative outcomes.

Variables	All Patients 253 (%)
Operation Side	
Right	203 (80.2)
Left	50 (19.8)
Operation technique	
VATS	208 (82.2)
Subxiphoid	37 (14.6)
Mini-thoracotomy	8 (3.2)
Complication rate of operation technique	
VATS	% 6.3
Subxiphoid	% 18.9
Mini-thoracotomy	% 25
Drainage time of operation technique± SD (days)	
VATS	2.8 ± 1.5
Subxiphoid	3.3 ± 1
Mini-thoracotomy	3.5 ± 2.1
Type of resection	
Upper-lower lobe wedge	204 (80.6)
Lower lobe wedge	16 (6.3)
Upper lobe wedge	14 (5.5)
Lower-middle lobe wedge	13 (5.1)
Upper-middle lobe wedge	3 (1.2)
Upper-middle-lower lobe wedge	3 (1.2)
Mediastinal lymph node dissection	3 (1.2)
Pleural biopsy	2 (0.8)
Complication	22 (8.7)
Drainage time ± SD (days)	2.9 ± 1.5 (Range: 1–15)
Mortality (30 days)	2 (0.8)

**Table 3:** Evaluation of pathology.

Pathological Diagnosis	Number of Cases (n)	Percentage of Cases (%)
Hypersensitivity pneumonitis	111	43.9
Idiopathic pulmonary fibrosis	45	17.8
Non-specific interstitial pneumonia	15	5.9
Usual interstitial pneumonia	4	1.6
Desquamative interstitial pneumonia	4	1.6
Organize pneumonia	5	2
Unclassifiable interstitial lung disease	8	3.2
Sarcoidosis	8	3.2
Cystic Lung Diseases *	7	2.8
Malignancy	6	2.4
Interstitial pneumonia with autoimmune features (IPAF)	4	1.6
Pleuroparenchymal fibroelastosis	4	1.6
Occupational lung diseases	3	1.2
Tuberculosis	2	0.8
Rheumatoid Arthritis	2	0.8
Churg Strauss Syndrome	1	0.4
Niemann Pick Syndrome	1	0.4
Pulmonary hypertension	1	0.4
Combined pulmonary fibrosis and emphysema	1	0.4
Idiopathic pulmonary fibrosis + Vasculitis	1	0.4
Non-specific group	20	7.9

* Cystic lung diseases include Brit–Hoge–Dube syndrome, lymphangiomatosis and pulmonary Langerhans cell histiocytosis.

**Table 4:** Comparison of selected studies evaluating surgical lung biopsy in interstitial lung disease.

Study	Year	Number of Patients	Surgical Approach	Complication Rate	Mean Drainage Duration	Diagnostic Yield
Khalil et al. [2]	2016	64	Surgical lung biopsy	12.5%	Not reported	89%
Zhang et al. [4]	2010	418	Surgical lung biopsy	10.5%	Not reported	91%
Lieberman et al. [8]	2017	45	VATS	11%	Not reported	91%
Durheim et al. [9]	2017	311	VATS	19.2%	Not reported	88%
Otsuka et al. [11]	2022	129	Surgical lung biopsy	9.3%	3.2 days	89%
Fibla et al. [13]	2012	311	Surgical lung biopsy	14.1%	2.5 days	87%
Ülker et al.	2026	253	VATS/Subxiphoid/ Mini-thoracotomy	8.7%	2.9 days	92.1%

## Data Availability

The data that support the findings of this study are available from the corresponding author upon reasonable request.
